# Understanding the difference, makes the difference: perceptions of Black and Minoritised ethnic occupational therapists on mentoring

**DOI:** 10.1186/s12913-023-10012-w

**Published:** 2023-10-02

**Authors:** Anita Atwal, Elizabeth McKay, Vimal Sriram

**Affiliations:** 1 School of Allied and community Health, London Southbank University, London, UK; 2 School of Nursing, Midwifery & Social Care, Edinburgh Napier University, Edinburgh, Scotland; 3 University Hospitals Bristol and Weston NHS Foundation Trust, Bristol, UK; 4 NIHR Applied Research Collaboration Northwest London, London, UK

**Keywords:** Mentoring, Minoritised, Workforce, Occupational therapists

## Abstract

**Introduction:**

Black and Minoritised Ethnicity (BME) occupational therapists face lower career progression opportunities and mentoring is one possible intervention that may offer support. BME persons may have different expectations and experiences from their mentor, and research exploring their perceptions of mentoring is important. In Occupational Therapy there is a growing awareness of the need to be true to the values of social justice. The aim of this research is to learn about BME occupational therapists’ experiences and perceptions of mentoring for career progression.

**Methods:**

Four online focus groups involving 19 participants from the UK were held, discussions were facilitated by researchers using a topic guide. Participants responses were analysed, and codes were brought together to create Themes including career progression and role models, mentoring relationship, similarity with the mentor and outcomes from mentoring.

**Results:**

The study highlighted that trust is integral to effective mentoring relationships and BME occupational therapists want mentors who they can trust with their experiences and stories. Compatibility between mentors and mentees in terms of personality, values, and working styles is crucial for effective mentoring relationships. Providing opportunities for networking, acknowledging good work and giving permission were all seen as outcomes from good mentoring. The absence of BME role models and ingroup bias were also reported as issues to be addressed.

**Conclusion:**

This study explores the perception of mentorship as a mechanism for career advancement in occupational therapists from BME backgrounds, with these results transferable to other health and care professional groups. We recommend the creation of a mentoring charter for BME healthcare workers in the United Kingdom to ensure that those from BME backgrounds feel supported, mentored, and provided with equitable access to resources, including adequate mentoring and networking opportunities.

**Supplementary Information:**

The online version contains supplementary material available at 10.1186/s12913-023-10012-w.

## Background

In Occupational Therapy there is a growing awareness of the need to be true to the values of social justice [1]. To do this we need to reflect on inequitable distribution of power and privileges to achieve fairness [2]. In the United Kingdom evidence suggests career opportunities and advancement for Black and Minoritised Ethnicity (BME) occupational therapists is lower than persons who identify as white British [3]. Mentoring is an intervention that is viewed as a mechanism to support career advancement, career progression and career plateau [4]. A meta-analysis [5] found that mentoring is associated with positive outcomes related to, attitudinal, health-related, relational, motivational, and career outcomes. In Occupational Therapy there is a paucity of research to demonstrate the effectiveness of mentoring on career outcomes and on workforce retention for minoritised groups. A concept analysis of mentoring in nursing defines the attributes of mentoring as – role model, nurturing, friendship, experienced person, regular meeting, and endurance [6]. A mentor is usually a more ‘senior’ person who takes an interest in the sponsorship of a more ‘junior’ person. The mentor often has expertise and experience and knowledge and want to see mentees achieve career success [7, 8]. There is also evidence that the quality of mentoring could influence workers intention to leave their place of employment [9]. Studies that compared non-mentored employees to employees with mentoring experience have shown more positive career outcomes [5].

The success of the mentoring outcome is dependent on human factors, in other words, the relationship between the mentor and mentee. Factors that may influence the mentoring relationship are both the mentor and mentee sharing similar demographic characteristics such as race, age and gender [10]. Findings from a study by Godshalk and Sosik [11] found that the greater the similarity between the mentor and mentee, the greater the likelihood for provision of psychosocial support, career development, and role modelling. If the mentor /mentee pairing is not compatible, trusting, close dyadic exchanges may not be able to be formed [12]. Evidence from the mentoring literature suggests that mentees who perceive themselves as being like their mentor’s, report more positive outcomes from their relationship than those who do not have these perceptions [5].

There are counter arguments in relation to the importance of mentor and mentee sharing similar demographics. Other reported variables impacting on the mentoring relationship are personality, values and working styles [13].Factors such as mentoring quality have been suggested as a means to mediate the effect of racial dissimilarity and access to sponsorship was viewed as more important than demographics [14, 15]. Reverse Mentoring is an example where senior white leaders (mentees) can learn from mentors (BME persons) about equality, diversity, and inclusion. There are also issues in this type of mentoring, it has been found to have greater benefits for the mentee than the mentor and unequal power dynamics of mentoring are sometimes reinforced [16, 17].

There is some evidence that BME persons may have different expectations from their mentor. An ideal mentor for white participants was someone who could coach them and provide feedback on career progression whilst persons from a minority background wanted an understanding mentor who provides empathy, guidance, and strong values [18]. Thus, research exploring perceptions of BME occupational therapists on mentoring is important since cross race relationships in Occupational Therapy are often the norm due to White therapists being the majority within the profession in the United Kingdom [19] We need to identify whether mentoring in its current format is viewed as a positive intervention. The aim of this research was to ascertain BME occupational therapists’ experiences and perceptions of mentoring for career progression.

## Method

This research is grounded within the principles of Participatory Learning and Action (PLA) as a research methodology in which all stakeholders are regarded as equal partners and collaborators in research [20]. It is anchored within the principles of co production and means that our research design needed to work with the research ideas and views that matter to BME occupational therapists [21]. PLA methodology is designed for research with diverse groups where asymmetries of power may exist [22]. We were particularly conscious of perceived differences in opportunities and experiences amongst different minority groups. We wanted an approach that would enable BME occupational therapists whose voices have not been heard within occupational therapy research. A PLA ‘mode of engagement’ promotes reciprocity, mutual respect, co-operation, and dialogue in research encounters within and across diverse stakeholder groups [22].

### Focus groups

Focus groups enable researchers to gain a rich understanding of the perspectives of a particular group of people by capitalising on the interaction that occurs within the group setting [23]. For some BME occupational therapists this may be the first time they have discussed their views on mentoring and career progression, therefore it was essential to create a sense of belonging and psychological safety. Online focus groups enable inclusivity since they remove barriers to travel and also are cost effective both in relation to finance and time [24]. Online focus groups allowed us to recruit from wide geographical areas which allowed us to gain a UK wide view of mentoring. In addition, we were able to reach BME therapists via social media who had not engaged with any affinity groups. It has been suggested that face to face focus groups encourage participants to discuss issues in greater depth. To overcome this in a virtual setting, we carefully limited the number of persons per group to encourage active participation. As the focus group discussed sensitive topics, we had two facilitators and an observer (the authors taking it in turns for each focus group session). The observer was responsible for the psychological wellbeing of any participants who may become distressed during the focus group. All participants were offered a de-brief after the focus group. We were concerned about the participants’ fatigue and wellbeing, so we ensured that the focus groups lasted no more than 90 minutes. To ensure psychological safety of participants post focus group support was available and offered. We aimed to have 5–6 persons in each focus group session.

### Participants

The dates and times advertised for the focus groups were planned to enable participants who were employed to attend, with a mix of day-time and evening times on different dates. Participants had to be Occupational Therapists (OTs) who identified as being Black, Asian or from a minoritised ethnicity background. They could be employed with the UK National Health Service (NHS), Local Authority, Voluntary or Independent Sector. In the UK, Occupational Therapists employed in the NHS are employed on the Agenda for Change [25] pay scale from Bands 5–9, where the higher banding usually signifies more senior roles. Some of the focus groups participants were recruited from those who completed a survey (*manuscript under review*) that explored mentorship in BME Occupational Therapists. The focus group questions (additional file 1) were constructed from the findings of this survey, a scoping review [26] and additional input from a steering group set-up to support this research. Participants received written information and a consent form electronically before the focus group, which were returned to the researchers. At the start of each focus group verbal consent from all participants was recorded. All focus groups were held over Microsoft Teams and were recorded with consent to enable transcription of focus group discussions.

#### Ethical approval

for this Study was obtained from London Southbank University Ethics Committee (ETH2122-0206).

### Analysis

In qualitative research using focus groups, analysis could be in relation to individual data, group data, and/or group interaction data [27]. In the focus group literature, there is no consensus as to the most appropriate unit of analysis for focus group data [28]. In our research we used the group and individual level data as the unit of analysis. We made notes in our own field notes information on group interaction data [27]. This allowed us to capture alternative voices that in turn increased the richness of the data and our understanding of BME occupational therapists’ perceptions and experiences of mentoring for career progression. [29]. To do this we utilised the constant comparison approach as suggested by Corbin and Strauss [30, 31].

Before analysing the transcripts, we discussed and reflected upon our own observations from our field notes and our thoughts about how the focus groups had operated. From the insights of these discussions, we noted themes, hunches and our own interpretations. The researchers worked together to analyse the transcripts and identify major themes by clearly describing our method of data analysis, providing clear evidence for our findings across the data set, and integrating our findings and interpretation [32]. The first step in analysis was to become familiar with the data and this included listening to the focus group recording multiple times; reading and re-reading of transcripts and field notes; and writing memos of initial ideas about the data. The second step involved open coding where we labelled chunks of data into small units. The researcher attached a descriptor, or code, to each of the units. Then, these codes were grouped into categories (axial coding). To do this we used the ‘long-table’ approach which required a comfortable large space. We followed the approach described by Rabiee [﻿^23^]. Prior to cutting the transcripts we (1) numbered each line of each transcript and ensured we had securely saved a full transcript (2) we did not use different coloured paper but gave each participant an identifying number and (3) we then started to arrange our working transcripts. Finally we developed themes that are specific to the content of discussions in the groups.

Reflexivity [33] and integrity of the research process was maintained by all authors. Two of the authors are from a BME heritage and in essence we found the focus groups difficult in the sense that many of the lived experiences were similar to our own. Focus groups did enable stories to be told, but on reflection we could have been better prepared for the raw emotion and the authenticity of respondents. To manage our own feelings, we did debrief after each session, however it made us even more determined to find solutions to ensure fairness in mentoring and career success. In addition, the authors’ experience as occupational therapists and health services researchers provided expertise necessary for this research. The authors had completed a scoping review on this research topic prior to the commencement of the focus groups [26]. The focus group question topic guide was informed by the results of this review, a survey, and discussions with the steering group (set up for this research study), as well as active participation from the participants during the focus group discussions, all of which assisted with triangulation. The authors’ personal and past experiences enabled us to conduct a qualitative study of this nature. It is acknowledged that authors’ previous experience may have influenced the coding and interpretation of the themes. All three authors conducted the focus groups and the authors met regularly to discuss and agree data collection, analyses, and interpretation.

## Results

We conducted four group sessions involving a total of 19 BME occupational therapists. Table 1 outlines participants in each focus group. All participants randomly picked a focus group date they found convenient to attend. Data from the Health and Care Professions Council [34] suggests that approximately only 8% of the occupational therapy workforce in the UK are from a BME background. Therefore, to protect the anonymity of participants, we have not presented location, level of seniority, detailed ethnicity details.

**Table 1:** Participant Characteristics

Focus Group	Participants		Age range	Work settings	Ethnicities from
	Male	Female			
1	1	5	22–65	Mental health, Secondary care	Asian Indian, Black African, Jamaican
2	0	5	30–59	Secondary care, Higher education, Mental health	Asian Chinese, Black African, Mixed-race
3	0	4	22–55	Mental health, secondary care, Independent	Asian Pakistani, Black African Mixed-race
4	1	3	30–59	Secondary care, primary care	Asian Pakistani, Asian Chinese, Black African

We analysed the data at both a group and individual level. Data saturation is usually used in qualitative research to ascertain sample size however there are differences as to when and how this is achieved. Evidence from a review of saturation in focus groups [35] suggests that 90% of saturation can be reached by conducting 4–5 focus groups. After four focus groups we did not perceive that additional new ideas would add further useful insights into mentorship for BME occupational therapists. We have used participants’ own words, with an understanding of the context (work environment, past experience, experience of mentoring) to illustrate the Themes whilst retaining the voice of occupational therapists’ personal experience and perspective illustrated through quotes from the focus groups. The analysis of the data from the focus groups is reported in four themes. These were Career progression and role models, Mentoring Relationships, Similarity with the Mentor, and Mentoring Outcomes. We have explained the themes in detail using quotes form participants with abbreviation of FG for the focus group in which the quote is from. Additional illustrative quotes are provided in Table 2.

**Table 2:** Themes with illustrative quotes

Themes	Focus Group 1	Focus Group 2	Focus Group 3	Focus Group 4
Career progression and role models	I agree that’s something we’re not very good at and why? … Why is it that we always feel like we have to play down things, you know, even when someone gives you a compliment… Is it the fact that we’ve been pushed down so much that we don’t believe in that ourselves? And perhaps that’s something we need to do to start raising our profile and to start feeling that we’ve got something to offer as well.	Mentor needed to have curiosity about your story.	They [white occupational therapists] cannot achieve it [career progression] and you know, they’re not BAME…how am I going to achieve it? It’s because it’s we have this perception that it’s 10 times harder for us to achieve things.	You can recognize it as marginalization and exclusion. how are we supposed to find ourselves, you know, belonging to our OT profession. I had a similar experience as well. There was a white clique who would all meet. I was not part of the gang.
Mentoring Relationship	You don’t need to be friends, but it is helpful if you are able to have that kind of authentic kind of relationship, We want to feel respected in a sense we don’t need to go out for a drink every day… We’re sharing a little bit of our personal lives … because you do connect to people more.	I don’t think we need to be best friends. We don’t need to be friends or associates, but as long as we’ve entered into that mentoring relationship with the same understanding having had that conversation about what that relationship is going to look like.	Someone you can trust and isn’t going to kind of palm you off because you’re not white.	I like the idea of them being kind of rated. I think that really helps in a sense of, you know, who else have you mentored?
Similarity with the Mentor	I think it feels like you’re in in good hands, it just needs [to be] a little bit more than a tick box exercise. if you had someone share their [life experience] examples and that’s great isn’t it? Because then they can’t fake it.	A mentor that is of same race with me should be able to understand certain cultural aspect of things. Like traditionally where I’m from in [country] we believe in professionalism, we believe in progress, we believe in achieving goals. It’s not about making the money or earning the salary… it’s about that progress, [it] is about achieving what you want to achieve.	I think it’s very important [to have a same race mentor], I think especially in the dynamic that I’ve recently had, there’s an older white woman and myself as a younger black woman. And my mentor named it in which was like, yes, ok you can do this [giving self, permission to discuss race issues].	Over the years [I] realise, it’s not everyone who looks like you, are for you. I’ve worked with people of colour that saw me as competition…not every single person who are of ethnic minority background is necessarily for [supporting] you
Mentoring Outcomes	It [mentoring] was acknowledged, it was worth the investment.	I’ve had mentors who have thrown my name into the circle. I’ve returned back from annual leave and all of a sudden I’m doing a talk for the [hospital] Trust board… I don’t think I would be where I am if that wasn’t the case.	From my experience it [being mentored] did have an outcome. I got what I wanted because at that point I didn’t know the culture of the NHS…and it was a culture shock to be honest…So I got what I wanted out of it. I’m still in touch with her today.	If I say something and somebody doesn’t think it’s important and someone else repeats what I say and everybody thinks it’s a fantastic idea, I’m still getting used to that by the way.

1. Career progression and role models:

Participants across the focus groups referred to the absence of relevant role models that they could look up to. This appeared to be rooted in the lack of available opportunities for BME occupational therapists and their confidence in themselves.

Occasionally decisions on workplace choice rested on representation and diversity.

*‘I got two [job] offers and I decided to go with the one [team] that was most diverse. It’s a conscious decision from me to seek out a workplace where the people [are] like me and that I’m able to have a mentor that looks like me’* (FG4).

Participants discussed issues around imposter syndrome, past experiences of achieving career progression being difficult and viewing achievements as routine.

*‘There’s something about what we need to do as Black women about our self-esteem and about the impostor syndrome. We’re brilliant, but often we don’t recognize that’* (FG1).

The participants wanted a role model and mentor that they could receive support from.

*‘I’ve never had a role model… especially in the NHS, there’s also like an identity thing where you’re not represented in those spaces. Oh, a person from the same race as me? what is that? What does that look like? Because it’s nothing that we’ve been able to experience*’ (FG2).

Others viewed having a mentor who looked like them had a better understanding of their experiences and were better role models.

*‘My mentor is a band 7 and she’s black and she, you know, she’s doing it … As a black person and you have to be conscious about where you go and OK and you see people that look like you there, is it [it is] possible to go higher in this organisation.*’ (FG4).

However, there was also recognition that just having a mentor from an ethnic background was not enough.

‘*Not every single person who are of ethnic minority background is necessarily for you [for mentoring]’* (FG2).

For other therapists, the work environment was impacting on well-being where they felt excluded or marginalised by other White occupational therapists.

*‘I have been crying a lot and I must try to control myself because, it’s so stressful. I work[ed] so hard to get my qualification and I never thought I was going to experience what I am experience [sic] now and especially being [in a] rotational [post]’ (FG3).*

This sense of being neglected was not restricted just to other White OTs, For some participants some BME occupational therapists in leadership positions had.

‘forgotten how they got there’ (FG1).

Others felt that there was little support from these professionals.

*‘[I was told] You should, basically stay in your lane… So I was expecting, so much support from my band six [agenda for change], who was also from the same cultural background. And it was such a difficult process because it was as if we’re competing… She was the only person of the BME community who was going to a band 7 [post]. So, I thought … this is clear progression here, but there wasn’t as much support [for me] as I would have thought*’ (FG1).

2. Mentoring relationship:

There was consensus that supportive work environments enabled ‘BME OTs to thrive’.

*‘Informal conversations are important as part of sharing life experiences’* (FG1).

and liking your mentor was not important but the relationship with the mentor was.

*‘It’s not so much about liking my mentor … You know being clear about what is it that we are, why are we in this relationship, what is the commitment I’m making and what are you expecting from me as a mentor’ (FG2).*

*The* relationship was most important which needed to be focused on trust. Authenticity and establishing Trust was seen as crucial, as the participants felt they needed a shared safe space to discuss troubling and often distressing issues.

*‘First, I think you need to establish trust and so it’s a safe space because we’d be sharing difficulties. So, it feels like you can share your weaknesses and that it is a space of growth and that you can learn from feedback. You’re both coming from the space of trust and security. And you know, I think if you don’t, it might feel like an attack. It might feel like a very exposing relationship. And I know you’re going to get hard messages’* (FG3).

Participants (FG2 and FG3) also felt,

‘White OTs did not like having difficult conversations about race’ (FG2).

Whilst also acknowledging that as BME occupational therapists they had to provide more support for others.

‘We need to ‘step up for other people as well’ (FG4).

There were also positive examples of allyship in mentoring conversations with White occupational therapists.

‘*So, I had an experience with one of the professionals I work with. I’m not quite sure they thought I had heard what they said under their breath. My colleague heard though, and he wasn’t having any of it. He just stepped up and said no. This is not happening this way. You know, like trying to tell what they did was wrong… The person that said the offensive word was shocked as well because they were not expecting to be checked. So that was an interesting situation’* (FG4).

There was a view that it was important to meet a mentor first,

*‘…to see whether a mentoring relationship could be formed and formulate a contract’* (FG1).

There was also a consensus across the focus groups that it was important to have the mentor’s profile available beforehand for the mentee to review.

*‘…so you could look at a mentors credentials and experiences*’ (FG4).

‘In my vision [sic] is over time you almost get a network and a community of trusted mentees. If someone did feel that they were worried about entering [into a] mentee relationship because of those dynamics, there’s maybe like a profile; this my experience, my previous mentees have endorsed me’ (FG 2).

Participants also felt that some mentoring could be provided as a group, especially to share experiences.

*I think I thrive off group work, just cause it’s bouncing off ideas . I think when it is one to one, sometimes I’m a bit on the spot and so I don’t like the pressure of that’ (FG3).*

Some participants also spoke about situations where the mentoring relationship had not worked.

*‘If you have a breakdown in a relationship which impacts the outcome of what you’re hoping to achieve…. I guess that’s where the line is. You know the integrity wasn’t there’ (FG2).*

‘…and I based it [establishing a mentoring relationship] on somebody’s profile… She is lovely person, but completely crazy. And I thought we were not going to get on at all. Just kept talking about herself. And that’s not what I’d want in a mentor, a crazy lady who did big research’ (FG3).

Overall, for a good mentoring relationship, skills that BME occupational therapists valued included listening (FG2, FG3); impartiality (FG1, FG2); prepared to challenge (FG4); understanding of the mentee (FG1, FG4); authenticity (FG2, FG4); integrity (FG2); and solidarity (FG1, FG2, FG4).

3. Similarity with the mentor.

Participants also discussed the need for similarity in appearance, understanding and lived experience between mentors and mentees.

‘*We’re speaking to power in the room and the potential disadvantage in the room’ (FG1)*.

For some participants it was important to have a same race mentor

‘They *(white mentors) don’t kind of get you in the same way that somebody from your background’* (FG3).

There was also a view that white occupational therapists did not like having difficult conversation about race, but some overcame these issues with frank conversations. Participants referred to this difficulty in having conversations around race issues as ‘naming it’.

*‘We can carry on with this mentoring relationship. She named it and said, you know what, what is this going to look like or how are we going to test the waters with the power dynamics here and sit with uncomfortable feelings. And how do we navigate and communicate that with each other? And I think by naming it, that put her also in a position of learning from me as well’* (FG1).

It was perceived that understanding each other in mentoring could overcome cultural differences.

*‘You don’t necessarily have to have someone that looks like them as their mentor, but you can have similar shared experiences even though you don’t look the same. If you understand each other and where you’ve come from and you’re able to speak up for each other’* (FG 4).

As mentioned previously in mentoring relationships, some participants had experiences where they felt that other BME therapists may not always be supportive in mentoring.

*‘Disappointed by placement educator who was the same race as me… And that was really disappointing because not only did I felt [sic] like they let down kind of….my kind of race but let down OT. And that was so disheartening’ (FG2).*

4. Outcomes of mentoring:

Mentoring outcomes should be seen in context of workplace pressures felt and articulated by BME Occupational Therapists. Occupational therapists had difficulties in accessing career progression opportunities such as secondment opportunities. Some even felt that speaking up about fairness could have a negative impact on career progression.’

*‘And sometimes when you speak up and you do name the [unacceptable] practice. I was branded a troublemaker. I have got some really lovely black, colleagues who still fight the fight. And we won’t shut up and be put up. But like it comes at a cost, and it comes at cost for your career, and that’s how that’s how I’ve experienced it, sadly’* (FG 3).

BME therapists acknowledged barriers to taking part in informal networks were social such as

*‘[other] Family commitments’* (FG1) and *‘going out [with colleagues] for drinks after work’* (FG2).

Building social networks were viewed as important to developing career opportunities.

*‘It was the network that supported me and colleagues in the network who you know, actually. Have you seen this [job opportunity]? it’s to try and develop the network and through my career it has been the network that has facilitated my progress. Like a lot of you [other focus group participants] there was no one encouraging me and signposting me for opportunities (FG1).*

Participants also identified varied factors associated with being a barrier to career progression, some related to limited representation at the workplace and or acknowledgement of presence including from other BME friends and colleagues, who could have acted as mentors.

*‘I applied to be an OT, you know, many people, Black people would say to me why are you applying. That is a white person’s job. So, I think it’s even harder as an OT because you are a minority in a profession.’ (FG2).*

Participants felt that mentoring of others was related to positive aspects of career development

‘*Experience of mentoring got me thinking outside the box on how to consolidate my skills, how to move forward (FG1)*.

Others felt that they gained from learning through their mentor about the mentor’s career progression.

‘*Mentoring was more about what did* you *learn on your journey that I could potentially use - skills and tools [for] coping on* my *journey. So, I think there’s something very important between mentor and mentee … I try not to do like [someone] said there. Well, I’ve done it, So can you’* (FG1).

participants also perceived mentors gave them opportunities.

*‘I have been fortunate to have people that’s kind of dropping my name [sic]… like my manager now has dropped my name…Yeah, you can walk around [with the] CQC [Care Quality Commission regulators] tomorrow. I’m like oh gosh, why is it me? But you know I guess it’s nice that they do believe in you’ (*FG4).

## Discussion

This is the first study that has explored mentoring experiences from the viewpoint of BME occupational therapists. Mentorship is thought to have two distinct functions (1) Career related - that included sponsorship, promoting exposure and visibility, coaching and protecting (2) psychosocial/personal development such as - role modelling, friendship, counselling [36]. Our findings articulate what BME occupational therapists want from mentors, mentoring relationships, and what impact this could have on their career progression.

We currently do not know the extent of the impact of mentorship on career progression for BME occupational therapists, but what is evident is that certain aspects of mentoring are particularly important for BME therapists. Our research has shown that if BME occupational therapy careers are to advance then we need to carefully reflect on what changes need to be made in the provision and facilitation of mentoring. Some of our participants reported an absence of BME role models in occupational therapy. The absence of BME occupational therapy role models is likely to prevent career aspirations from being achieved. We can assume that current programmes of supportive mentorship are not fit for purpose, if they ignore the experiences of BME occupational therapists in professional practice.

It is evident from our research that relationships are critical within cross race mentoring process. We know that mentoring requires trusting, close dyadic exchanges between mentors and mentees [12]. What is a new finding from our research is that mentors need to be comfortable listening to and gaining the trust of BME occupational therapists. Trust has not been explored sufficiently in the mentoring literature [37]. Our findings suggest that to build trust, mentors and mentees should spend time deciding whether this relationship could work. In essence this is particularly important in cross race mentoring since social identity theory suggests that characteristics such as ethnicity, gender and class are used to develop relationships and a sense of connectivity [38]. Two meta-analyses found that mentees who perceived themselves to be similar to mentors, report positive mentoring outcomes [5]. Findings from another study [11] found that the greater the similarity between the mentor and mentee, the greater the likelihood for provision of psychosocial support, career development, and role modelling. If mentee and mentors are not compatible in relation to personality, values and working styles, this can cause mentoring relationships to be ineffective [13] and that both mentor and mentees need to agree on how to manage issues related to cultural and social differences. All of which is reflected in our study findings for BME occupational therapists and could potentially be transferred to other professional groups in health and social care.

Key skill for a mentor that have been identified in the literature are the willingness to share personal and professional experience and to act as advocates for mentees [39].Black medical students suggest that racism needs to be addressed within ‘cross cultural mentoring’ [40]. Our research adds to this, mentors in cross race relationships need to be prepared to listen to the mentees’ race related experiences and should be an aspect for consideration within mentoring.

I﻿n our study some participants also felt that some BME role models may not be supportive and/or an ally. An ally is someone who supports the social identity of marginalized groups and fights injustices against mentees and is willing to look beyond the initial stages of allyship to foster long lasting mentoring that becomes sponsorship [41]. Sponsorship is an important part of allyship and in order to enable careers to progress. In our study BME occupational therapists were less focused on friendship but wanted a mentor who they could trust with their experiences and stories. Our findings support those of Bailey-Johnson [18] who found that Black students unlike white students wanted a mentor who provided them with empathy, guidance and strong values. Within occupational therapy it is important to explore in more depth ingroup bias which leads to viewing a member of one group more positively than others [42]. Informal socialization systems refer to those relationships and contacts that facilitate access to career and social support, informal social networks are considered to add more value than formal networks in the accomplishment of both organisational (macro) and individual (micro) objectives and goals for career progression [43]. However, for Black women these opportunities may be less accessible than for White women [44].

Based on our findings, we would recommend the creation of an Athena Swan type charter for mentoring for BME healthcare workers. The Athena Swan Charter is ‘an international framework developed to support and transform gender inequalities and embedding inclusive cultures that is administered by Advance HE within higher education and research’ [45]. The charter assists institutions to meet equality legislation requirements, as well as the requirements and expectations of some funders and research councils in the UK. The Athena Swan charter uses a targeted self-assessment framework to support applicants (departments or institutions) identify areas for positive action as well as recognise and share good practice. Athena SWAN has been found to be a catalyst for improvements in institutional engagement and communication around diversity practices after receiving an Athena award [46]. It gives a clear indication of the level of commitment and or outcomes achieved with institutions awarded a gold, silver or bronze award.

We would recommend institutions that employ health and care professionals create and adopt a similar charter in ensuring that those from BME backgrounds feel supported, mentored and provided with equitable access to resources including adequate mentoring and networking opportunities. We recommend institutions such as the Royal College of Occupational Therapists, NHS Race and Health Observatory, Medical Royal Colleges and other major research funders and voluntary sector organisations champion such a move to create this charter. We are in the process of co-producing a new model of mentorship with BME occupational therapists. What is evident is that already known aspects of mentoring such as building relationships, sponsorship and trust must be achieved within cross race mentorship relationships. There needs to be further co-production work to reflect on allyship within cross race mentorship and how issues related to racism are managed.

### Strengths and limitations

This is the first study that explored the perceptions of BME occupational therapists on mentoring for career progression. Our research only included BME occupational therapists in the UK and there is a need to capture the voice of White occupational therapists to ascertain what their (mentors and mentees) perceptions of mentoring BME occupational therapists is like. We captured a wide variety of occupational therapists in the UK from different health and care settings, but the narratives from those in independent sector employment is less, despite our continuous efforts to identify and invite them to participate in the focus groups.

## Conclusion

This study explored perceptions of BME occupational therapists on factors that hinder and or advance mentoring relationships. These findings will likely resonate with other health and care professional groups and hopefully acts as a beacon call to inspire additional support and equitable access to mentoring and career progression for other minoritised health and care professionals. Due to current professional demographics cross-race mentoring is likely to continue, however there needs to be a reflection on how supporting mechanisms are put in place to manage racial and cultural differences during mentoring, which thereby may impact on career progression and additional role modelling. Relationships and trust are critical to this process and without trust and allyship mentoring outcomes may not be achieved.

### Electronic supplementary material

Below is the link to the electronic supplementary material.

Supplementary Material 1

Supplementary Material 2

## Data Availability

The datasets generated during the study are available from the corresponding author on reasonable request.

## Declarations

### List of abbreviations

BME – Black, Asian and Minoritised Ethnicity.

NHS – National Health Service.

OT - Occupational Therapy.

UK – United Kingdom.

### Authors’ information

AA, EM and VS are Occupational Therapists. AA and VS identify as BME. All authors have worked in healthcare and academic settings and have experience in research methods and have mentored occupational therapists and other health and care professionals as part of their career.
