# Supplementary material for: Understanding the difference, makes the difference: perceptions of Black and Minoritised ethnic occupational therapists on mentoring
Source: BMC Health Serv Res. 2023 Oct 2;23:1050. doi: 10.1186/s12913-023-10012-w (PMC10544464; doi:10.1186/s12913-023-10012-w)
Supplement: Supplementary file 2 — Supplementary Material 2 [file 12913_2023_10012_MOESM2_ESM.docx]

Topic Guide for focus groups:

*Pre-focus group:*

*Participant to re-receive the information sheet and asked to read it through. Participant will be given a brief introduction to the research.*

- *Introduce researchers and debriefing if anyone is distressed.*

*• Participants to be told what will happen during the focus group and reminded that the focus group will also be recorded.*

*• Participants to be told that an anonymised transcript will be made from the audio recording.*

*• Participants to be told the method of analysis and reminded that they will remain anonymous, and that their data will be confidential.*

*• Participants given time to ask questions*

**Focus Group:**

1. Can you tell us a bit about yourself and your current situation?
2. Describe your mentoring experiences?
3. What would you look for in an Ideal Mentor?
4. What is important in mentorship (prompt - the skills of a mentor and or demographic factors of the mentor such as race, gender)?
5. Do same race and or cross race relationships differ within the mentoring process?
6. What is a safe space? What should it look like?
7. How would you build a mentoring programme for BME OTs?
8. Should white mentors have reverse mentorship prior to mentoring a BME Occupational Therapist
9. Should relationship building happen before the mentoring process?
10. How do you know if mentoring is worth the investment?
11. Is there anything else you would like to add about your mentoring experience?
12. Summary and any other questions.
